# On Apoplexy

**Published:** 1802-07

**Authors:** 


### *To the Editors of the Medical and Phyfical Journal.*

Gentlemen,

The difcuffion which has lately occupied your pages on the fubject of Apoplexy has been now protracted to a length, which may poffibly induce you and fome of your readers to wifh that it may not be further extended, left it fhould operate to the exclufion of other fubjects, equally deferving inveftigation, and be liable to make your publication a vehicle of acrimonious controverfy, rather than of ufeful knowledge. But it fhould be recollected, that on many points the parties are ftill at iffue; and we may reafonably indulge the hope, that, by a collifion of opinion, fome ufeful information may ultimately be drawn forth, for the benefit of medicine.

Under this impreffion I take leave to offer a few remarks, not with the expectation of adding to our knowledge, but from a wifh of having what is already known fairly appreciated. The fceptical mode of proceeding adopted by your acute correfpondent Pyrrho, in your 38th Number, is not, in my judgment, haftily to be flighted, as the fuggeftion of doubts, upon any fubject, may ferve to reprefs too great reliance upon authority, may lead to a fuller inveftigation, and probably to a detection of error, which is one great ftep towards the attainment of truth.

In the different communications upon this fubject, two points appear to have occupied attention, the caufe and the treatment of Apoplexy; and as the latter will neceffarily depend upon the former, it is of importance that a correct opinion, as far as poffible, fhould be formed. I do not revert to the origin of this difcuffion difcuffion, further than to obferve, that Dr. Langflow, whofe talents I will venture to pronounce refpectable, has furely given his fentiments in a ftile of confidence, fomewhat inconfiftent with fcientific purfuits, and which cannot be juftified from the imperfect ftate of medical knowledge.

Admitting that extravafation or effufion takes place in every cafe of true Apoplexy, it is incumbent upon us to determine, if poffible, whether that be a caufe or effect. " Ut non plethora, ad eofdem excitandos morbos, fic neque fanguinis aut feri fuper cerebrum effufio, pertinet. Hæc fatis quidem frequens eft, fed ultimus eventus, non prima caufæ origo. Quin et fimilis effufio omni vaforum ex magna debilitate laxitati communis fubeft." When we reflect upon the time of life and the mode in which fuch a difeafe ufually attacks; upon the apparent ftate of the fyftem, combined with other concomitant circumftances, I think it may be fufpected that thofe appearances difcovered on diffection, may be regarded as confequences only, and that the common practice of copious bleeding is at variance with legitimate theory. Are not the difeafes which attack people at the time of life when Apoplexy ufually comes on, for the moft part, difeafes of debility? Do not moft of the fymptoms connected with Apoplexy, indicate a want of power in the fyftem? Are there not alfo abundant proofs, that at the fame period of life, the energy of the brain, and probably of the veffels of the head, is much diminifhed, and upon the decline in point of power? Although the veffels of the head fhould have given way, it is not neceffarily connected with increafed vafcular tone, either general or local. Is it not more probable, as a fair inference, that from a degree of weaknefs pervading their fubftance, they have received a larger portion of blood than they can propel, and yield to preffure and diftenfion? Suppofing there be any probability in this fuppofition, what is the mode of practice which it fuggefts? To render the circulation of the blood, which is probably accumulated in a particular part more equal, by the judicious ufe of internal and external ftimuli, which are calculated to excite the action of the nervous fyftem, and thereby to roufe the depreffed powers: To unload the bowels by irritating means, which will not only quicken the circulation in their veffels, but as the torpor which commonly prevails in fuch habits often affects the bowels, that accumulation of fæces, which tends to deprefs and greatly to impede the functions, fhould be removed with all diligence. Local bleeding fhould not be excluded.

In taking this view of the fubject, it will be known to many from whence the ideas are borrowed; and as they feem to have been fomewhat overlooked in this difcuffion, I have referred to them in the hope of deciding by the experience and obfervation of of any of your correfpondents, who may be induced to notice thefe remarks, to what extent they can be purfued and fupported.

In regard to emetics, it does not appear, notwithftanding the authorities which have been quoted, that we have fufficient experience to juftify their being reforted to as a remedy in Apoplexy. I am fpeaking here of the genuine difeafe, occurring, as is generally the cafe, when life is advanced. In flight attacks, and where there is rather a predifpofition exifting than a full-formed difeafe, they may fometimes be adminiftered with good effect, fhould the ftate of the ftomach require fuch a remedy; but might there not be fome danger, in a fevere difeafe, independently of either violently impelling the blood into the head, or impeding its return, of overwhelming the remaining and labouring powers of the fyftem by fo urgent a ftimulus? For in defiance of all the reafoning and refinement that may be adopted, I acknowledge myfelf miftaken, if full vomiting, in a full, plethoric habit, the fact itfelf being fairly adhered to, do not urge unduly the vafcular fyftem, and endanger determination and congeftion, efpecially in parts already prone to difeafe. But the merit of fuch remedies muft be decided by a fair trial, and I have yet to learn that the practice of the prefent day, from repeated proof, has fanctioned their ufe; but in referring to *modern* practice, I by no means intend to depreciate the merit of thofe who have gone before us; who have, by their exertions and various abilities, contributed to lay the foundation of much of that knowledge which we poffefs; but I fufpect we are more indebted to them for valuable facts than for any theory, on which practical rules can be founded.

In propofing thefe remarks to your confideration, fhould you think them not undeferving a place in your Journal, I feek for information, and fhould be glad, by the affiftance of others, to have the opinions which I am inclined to efpoufe, either confirmed or refuted; that in the moment of exigency, the mind may have fomething to reft upon, and efcape that doubt and diftraction which too often embarrafs it, in purfuing the medical profeffion.

I have obferved, that by fome of your correfpondents, fpafms and convulfions have been mentioned as occurring in fome cafes of Apoplexy. Cullen ranks that difeafe under the clafs Neurofes, part of the character of which is fpecified by "fenfus et motus læfi;" and in the definition of Apoplexy which commences with "motus voluntarii fere omnes imminuti," there is not a word of convulfion or fpafm; has there been no error in the diagnofis in fuch cafes? And do not apoplectic fymptoms fometimes occur in cafes, without genuine Apoplexy?

